# Pulmonary Congestion and Its Treatment. II

**Published:** 1895-05-18

**Authors:** Benjamin Ward Richardson


### Pulmonary Congestion and Its Treatment.—II.

By Sir Benjamin Ward Richardson, M.D., F.R.S.

(*Continued from page* 57)

I indicated in the last paper two striking facts of a clinical nature, recording a case of pulmonic congestion in which I waited before drawing blood, and witnessed what might be called natural recovery without resorting to that measure. I adduced another case in which the congestion being more extreme, and being attended with great pain from pleuritic friction, free venesection gave immediate relief even to an emaciated man. I showed that the blood changed in colour as it was being drawn, becoming redder in character, a fact that has been observed by Hippocrates, and that indicated a better oxidation of the blood in consequence of a freer circulation through the lungs. I also showed that pain was relieved, but omitted to mention that this fact of subsidence of pain during abstraction had been noticed as a fact by the great Galen, who once had himself bled in order to be relieved of pain, and with success. The question in this day is, why we should hesitate to bleed, and why did I, who once would have bled without a moment's pause, hesitate to draw blood, and wait for the development of more extreme symptoms. Had there been pain of an acute kind I should not have waited, but there is no concealing the fact that the present aversion to the extraction of blood had an effect upon my mind which would not have been felt in former times. What we want now to understand is the precise moment when to draw blood in such instances as those before us. We cannot do it at present with impunity, that is the broad fact, and our knowledge is not so precise as to make us sure when we can do it without discredit to our skill, or injury to our conscientiousness. We resort, therefore, to minor measures, and wait, at the risk of secondary consequences, for the course which the disease will take. Sometimes, as we have seen, the congestion takes a natural course, at once towards cure, sometimes it does not. I think as a rule it does not. What then, as minor proceedings, are the best things to do? My own line of action is clear and defined, and I hope it is scientific as well—at all events it is very successful, which is satisfactory. First of all, I lay the patient down in the horizontal position as far as it can be done, because that position relieves the heart of considerable labour. When the body is erect the volume of blood which goes over both the great and the little circulation must traverse those circuits against gravity. When the body lies down the circulation is in the horizontal course which must be of benefit. Next, I take particular care to keep the body warm by covering it with light, porous clothing, and paying especial attention to the fact that the feet and lower extremities are kept specially warm. Again, as far as possible, I have the body carefully fed, content to use light nourishing foods of a liquid character. Milk appears to me to be the best food that can be given, but it is well to dilute it with warm water in equal parts, for the simple reason that it appears to digest better when used in that way. I have very often had the milk peptonised, and I really think there are instances when that process seems to be an advantage, for there are persons who have lost the power of digestion of milk, as if the digestive principle of milk connected with the infantile state had ceased to exist, and as if the peptonising plan met the difficulty. If milk cannot be taken, eggs very often can, and eggs are easily made up, by good manipulation, so as to form a thin fluid which can be admixed with a little milk and water and can receive a fruit flavour. At the same time I allow fruits to be taken such as grapes, grated apple, or grated pear, or the juice of an orange, in small quantities. A very nice dish can be made by boiling grated apple, all indigestible parts being removed, in thin sago. On this simple diet patients subsist perfectly well during even a prolonged attack. Of drinks I avoid all of alcoholic kind. I am sure alcoholic drinks do great mischief. They do not support the body; they cause relaxation of vessel; they excite the heart, enlivening it to an action which is quick, feeble, ineffective, and at the same time exhaustive; while they also excite the brain so greatly that the enfeebled patient becomes more or less intoxicated and unreasonable. I do not hesitate to say that more than once I have found a patient treated with alcohol actually intoxicated, and have had the phenomena pointed out to me as an aberration of mind, a delirium resulting from the disease and not from the remedy; a diagnostic mistake which, I believe, occurs in various diseases and on frequent occasions. Tea, except in small quantities, is not allowed in these cases; but a very good fluid, which patients like, and often call reviving, is good coffee treated with small doses of carbonate of ammonia, with a little sugar to make it agreeable to the taste. One or two three-grain tablets of carbonate of ammonia dissolved in eight ounces of coffee, with two drachms of sugar, makes an exceedingly nice beverage, and can be taken either warm or cold, if it be carefully strained. Some persons will say that in so administering ammonia a stimulant is being introduced as if it took the place of alcohol. The mistake is grievous. Ammonia does not act at all in the same way as alcohol. It constricts rather than relaxes vessels. It acts favourably in promoting excretion by the glands; it is antiseptic, and, above all things, it tends to keep the blood fluid, and so prevents the formation of large or small clots of fibrine in the heart and in the blood vessels. Medicinally, I use, I may say, invariably, two remedies, ammonia and digitalis, the formula I most like being five minim doses of tincture of digitalis, five grains of carbonate of ammonia, and two fluid drachms of liquor ammoniæ acetatis, the dose to be given every three to six hours with four ounces of distilled water. These measures, with the temperature of the air at 64 deg. Fahr., and with not more than 60 deg. of moisture is, I believe the best treatment; and, in my observation, the most successful.

